# Endovascular repair of abdominal aortic aneurysm with severely angulated neck and tortuous artery access: case report and literature review

**DOI:** 10.1186/s12893-015-0005-5

**Published:** 2015-03-08

**Authors:** Qinglong Zeng, Lianjun Huang, Xiaoyong Huang, Mingliang Peng

**Affiliations:** Aortic Center of Anzhen Hospital affiliated to Capital Medical University, Beijing, China

**Keywords:** Abdominal aortic aneurysm, Hostile neck, Endovascular aneurysm repair

## Abstract

**Background:**

Endovascular aneurysm repair has revolutionized the therapeutic strategy for abdominal aortic aneurysm. However, hostile proximal aneurysmal neck and tortuosity of access vessels remain challenges in selecting optimal stent-grafts in abdominal aortic aneurysms with difficult anatomy.

**Case presentation:**

A 65-year-old woman complained of intermittent abdominal pain for one week. Computed tomography angiogram demonstrated a tortuous infrarenal abdominal aortic aneurysm with a tapered neck and a 136° of infrarenal angulation. Aneurysmal dilatation and severe calcification of bilateral iliac arteries and tortuous aortoiliac access were also showed. Endovascular approach using Endurant stent-graft was attempted at an outside hospital, but failed because of the significant tortuosity of the abdominal aorta and iliac arteries. Since the patient refused to have open aneurysm repair, he was transferred to our hospital for further evaluation and possible EVAR with a different approach. EVAR was performed successfully using Gore Excluder stent-grafts (W.L. Gore & Associates, Flagstaff, AZ, USA). During the procedure, cannulation of the contralateral limb was unable to be achieved because of the tortuous aortoiliac course. Therefore, a snare was inserted from right radial artery, through the contralateral gate, to grasp the wire from left femoral artery. Two iliac stent-grafts were sequentially deployed with the lower end distal to the opening of the left internal iliac artery. Angiography confirmed complete sealing of the aneurysm with patency of bilateral renal arteries and external iliac arteries. The postoperative courses were uneventful and follow-up computed tomography angiogram at 6 months demonstrated patent bilateral femoral and renal arteries without endoleaks or stent migration.

**Conclusion:**

Although endovascular repair of aortic aneurysm with hostile neck and tortuous access is rather challenging, choosing flexible stent-grafts and suitable techniques is able to achieve an encouraging outcome.

## Background

Endovascular aneurysm repair (EVAR) has revolutionized the therapeutic strategy for abdominal aortic aneurysm (AAA) with low operative mortality and morbidity, short hospital stay, and minimal blood loss compared with open repair [1,2]. The advances of endovascular technology and learning curve effect of endovascular specialists have gradually expanded EVAR indications for AAA, even in the aneurysms with unfavorable anatomic morphology [3-6]. However, unfavorable proximal neck and hostile artery access still be challenging to vascular surgeons [7,8]. In addition, optimal selection of stent-grafts in EVAR remains unknown [9]. We report an EVAR case with severely angulated neck and hostile artery access treated successfully with Gore Excluder stent-graft system.

## Case presentation

**Figure 1:**
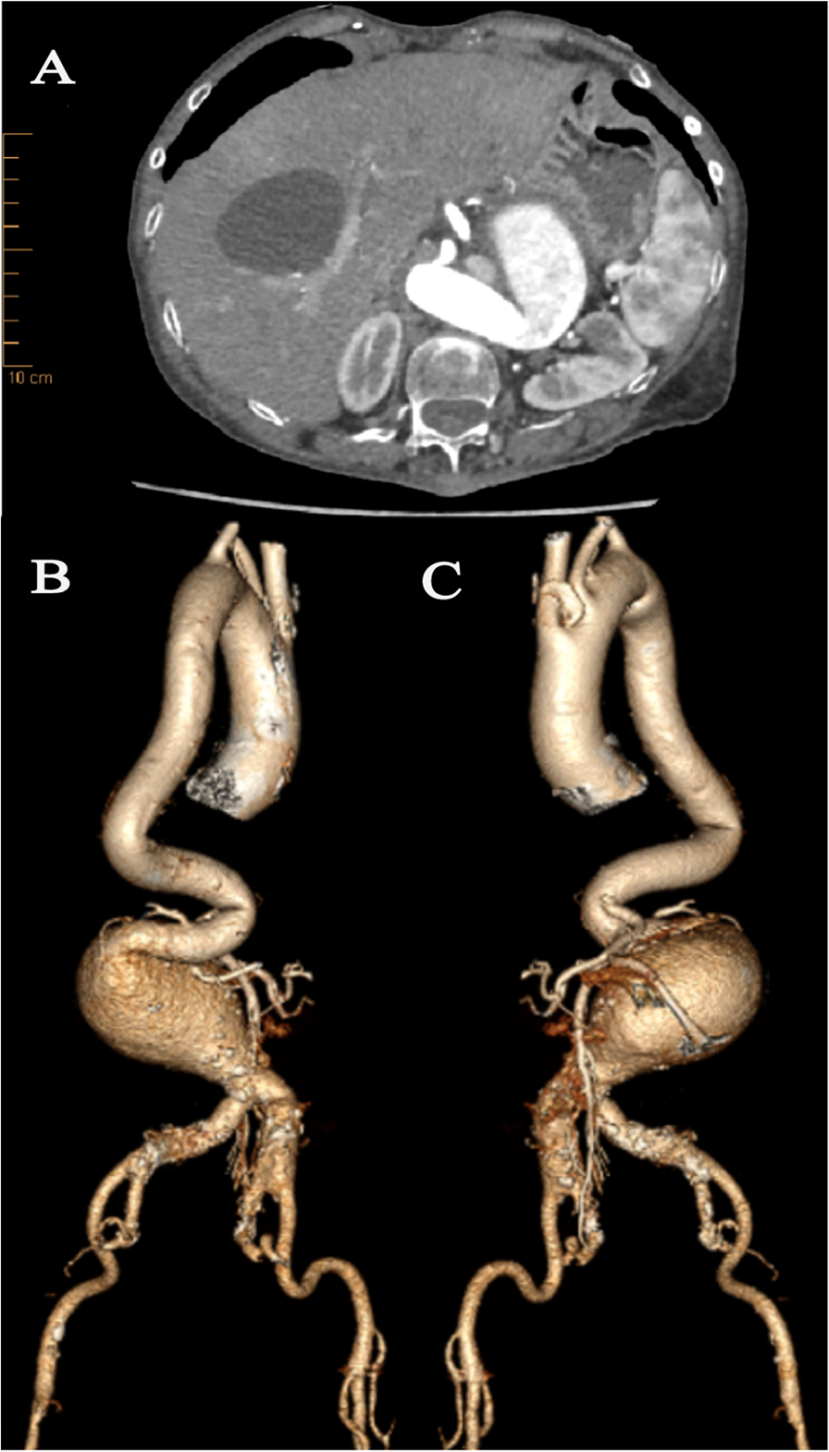
**Preoperative CTA and 3-dimensional reconstructions. (A)** Preoperative CTA showed the infrarenal AAA with tapered neck and severe angulation in axial view. **(B, C)** 3-dimensional reconstructions confirmed severely tortuous descending aorta and infrarenal AAA with really hostile neck. Aneurismal dilatation and severe calcification of bilateral iliac arteries and tortuosity of left aortoiliac access were observed.

A 65-year-old woman presented to an outside hospital with complaining of intermittent left-lower quadrant abdominal pain for one week. Computed tomography angiogram (CTA) demonstrated a severely tortuous descending thoracic aorta and an infrarenal AAA with maximum diameter of 80 mm and hostile neck (23 mm length neck with proximal diameter of 20 mm, distal diameter of 13 mm, maximum diameter of 23 mm and a 136° of infrarenal angulation) (Figure 1A,B). In addition, aneurysmal dilatation and severe calcification of bilateral iliac arteries with a 17 mm and 23 mm diameter on the left and right respectively, and tortuosity of aortoiliac access were also showed. An endovascular approach using Endurant stent-graft (Medtronic Vascular, Santa Rosa, CA, USA) was first attempted through left femoral access at an outside institution, but failed to angiographically confirm proximal neck after introducing delivery system below the origin of renal arteries because of the significant tortuosity of the abdominal aorta and iliac arteries. The patient was then transferred to our aortic center for further evaluation and treatment.

Previous medical history included a hypertension, stroke with residual left limbs weakness. The patient was diagnosed to have AAA by CTA with maximum diameter of 40 mm in 2012, but didn’t receive regular follow-up. Due to the underline comorbidities and patient’s refusal to open surgery, a second EVAR was carried out after consent was obtained. We decided to use Excluder stent-graft system which is more flexible based on our previous experiences.

**Figure 2:**
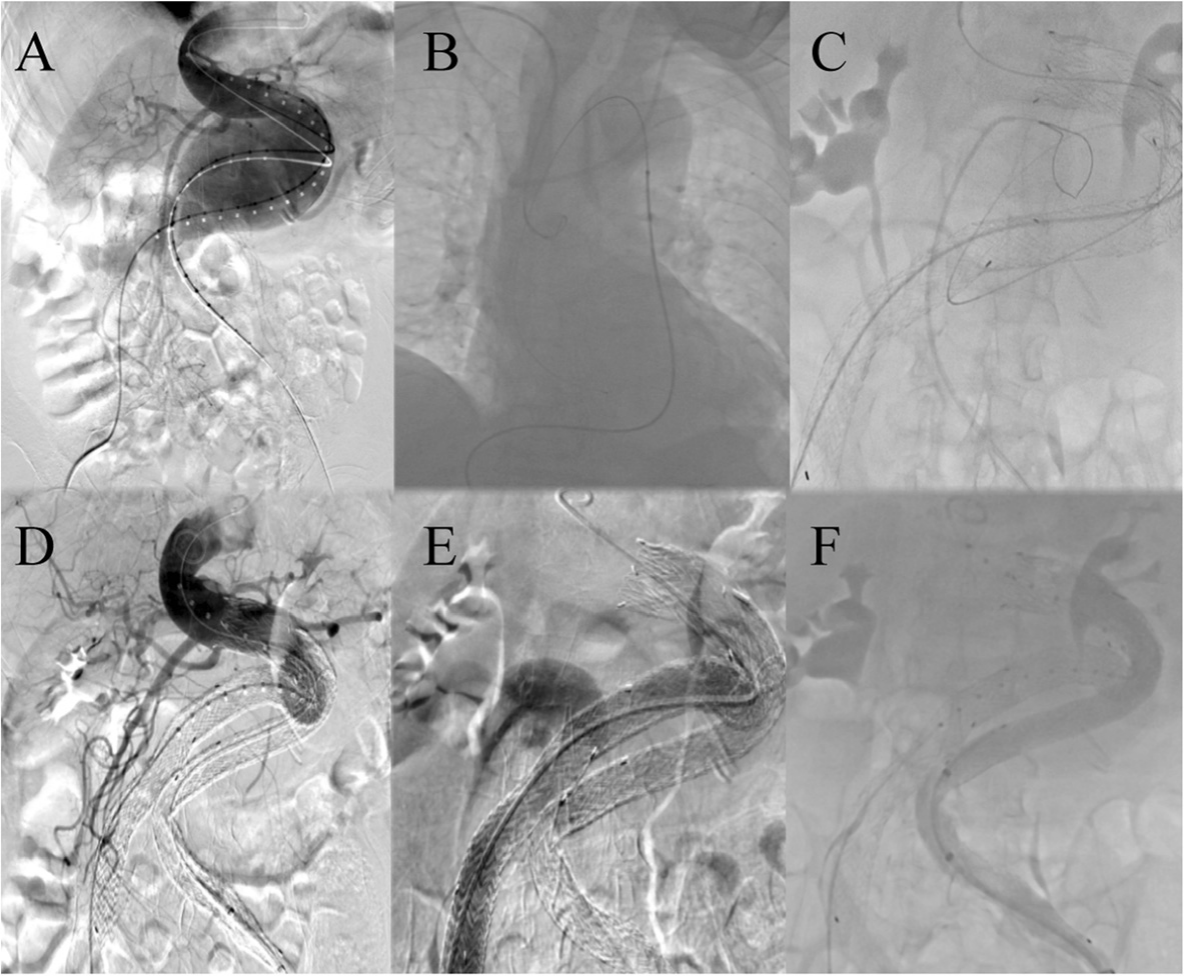
**The angiography during the procedure. (A)** Diagnostic angiography demonstrated the presence of unfavorable infrarenal AAA. **(B and C)** The snare sequentially caught the super smooth wire in ascending aorta and outside left iliac leg to build the left iliac access. Angiography confirmed complete sealing of the complicated AAA with fluent bilateral renal arteries **(D)** and iliac arteries **(E and F)**, but delayed visualization in left iliac artery.

**Figure 3:**
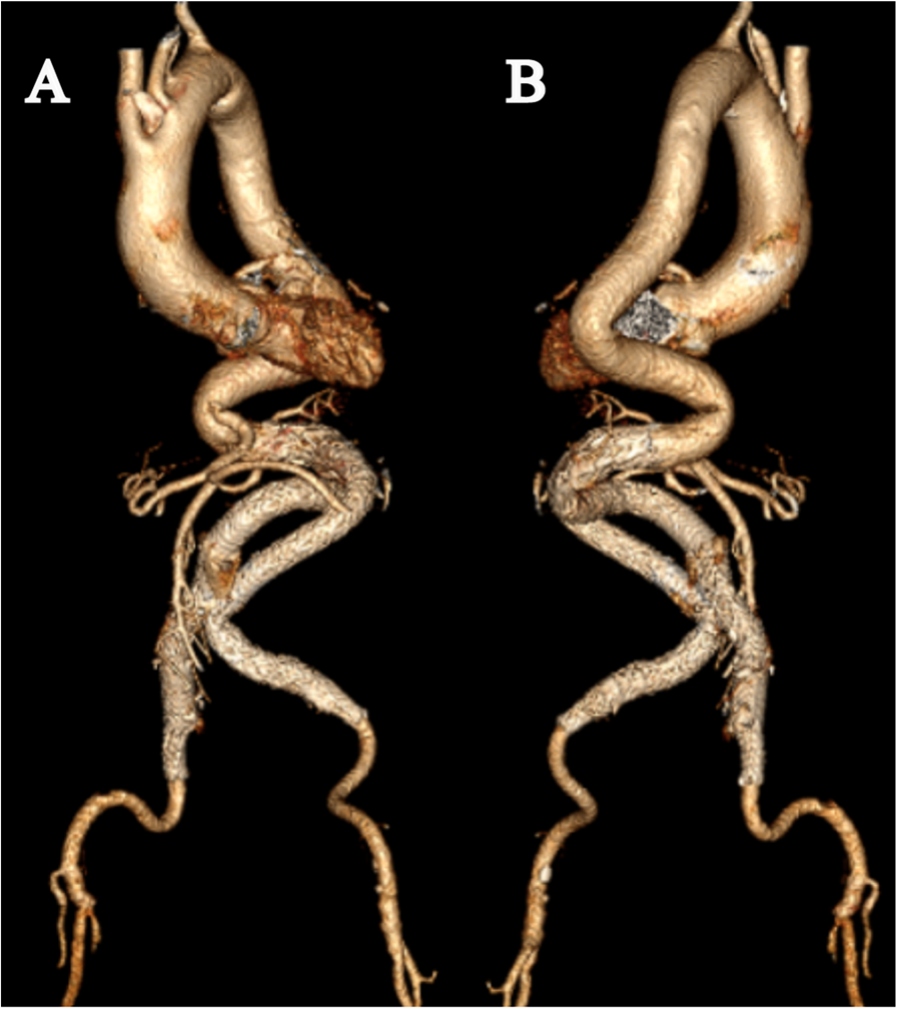
**Anterior (A) and posterior (B) view of 3-dimensional reconstructions at 6 months follow-up showed favorable stent-grafts positions and continued perfusion of both femoral and renal arteries.**

Following intravenous sedation and local anesthesia, two pigtail catheters were introduced through bilateral femoral arteries respectively after surgical exposure. Diagnostic angiography confirmed the presence of unfavorable infrarenal AAA (Figure 2A). An 18 F sheath (W.L. Gore & Associate, Flagstaff, AZ, USA) was introduced from right femoral artery and an Excluder PXT 281418 aortic main body (W.L. Gore & Associate, Flagstaff, AZ, USA) was deployed below the origin of the lowest renal artery over the Amplatz super stiff guidewire (Boston Scientific Corp., Marlborough, MA, USA). Two iliac stent-grafts of PXL121400 and PXL161407 (W.L. Gore & Associate, Flagstaff, AZ, USA) were successfully placed with the lower end distal to the opening of right internal iliac artery. But cannulation of the contralateral limb was unable to be achieved because of the tortuous aortoiliac course. Therefore, an Amplatz Goose Neck snare (EV3, USA) was inserted from right radial artery to catch the super smooth wire (ASAHI INTECC, Japan) in ascending aorta, through the contralateral gate, to grasp the wire from left femoral artery (Figure 2B,C). Two Excluder PXL141400 iliac legs (W.L. Gore & Associate, Flagstaff, AZ, USA) were sequentially deployed distal to the opening of the left internal iliac artery. Angiography confirmed complete sealing of the complex AAA with patency of bilateral renal arteries and external iliac arteries (Figure 2D,E,F). The procedure time was 1.5 hours. The patient’s recovery course was uneventful and she was discharged on postoperative day 3. She was symptom-free without claudication and serum creatinine was 79.0 μmol/L. Follow-up CTA at postoperative 6 months demonstrated favorable stent-grafts positions without endoleaks or migration. Bilateral femoral and renal arteries were patent (Figure 3A,B).

## Discussion

EVAR has been widely used as an alternative to open repair for patients with AAA since it was first reported by Parodi in 1991 [10]. However, unfavorable morphology of aneurysm especially hostile proximal neck has restricted the widespread application of EVAR. In a meta-analysis, hostile neck was defined as conditions that were not suitable for use of engdograft devices employed in the selected studies, which included anatomical factors such as neck length <15 mm, infrarenal neck angulation > 60°and neck calcification or thrombus covering > 50% circumference of aortic diameter and a reverse taper morphology [11]. Interestingly, growing recent studies showed feasibility and safety of EVAR in patients with hostile neck, but the anatomical indications of outside EVAR instruction for use (“off-label use”) reach no common consent [4,12,13]. Given the need of intervention and patient’s refusal to open surgery, an endovascular approach was performed despite severely angulated neck and hostile artery access in our case.

Disputes still exist in the efficacy of EVAR in patients with hostile neck. Stather et al. reported a largest study to date investigating the effects of neck angulation, neck diameter, neck flare, and neck thrombus on outcomes following EVAR, and contained the mean follow-up 4.1 years. They came to the conclusion that hostile neck anatomy could be successfully treated with EVAR. However, surveillance was necessary to detect and treated late type I endoleaks in hostile neck patients [3]. Antoniou et al. systemically reviewed the studies compared the outcomes of EVAR in patients with hostile and friendly neck anatomy. They found that although patients with hostile neck had no significant increased early incidence of type I endoleak and re-intervention rates, EVAR in patients with hostile neck required more adjunctive procedures to achieve proximal seal compared to the patients with friendly anatomy. Furthermore it resulted in a twofold increased risk of 30-day morbidity, a fourfold increased risk of type I endoleak and a ninefold increased risk of aneurysm-related mortality within 1-year follow-up. They finally suggested that EVAR should be cautiously used in patients with hostile neck [11]. The evidences from European Collaborators on Stent-Graft Technique for aortic aneurysm repair database demonstrated a significantly higher incidences of proximal neck dilation, proximal endoleaks and need for secondary interventions in patients with severe (>60°) infrarenal angulation [14]. But a morphological study about remodeling of proximal neck angulations of AAA after EVAR showed a larger angulation reduction and a smaller diameter shrinkage of AAAs with a neck angulation >60° in a mid-term follow-up, and concluded that proximal neck angulation was not a major issue in AAAs with adequate proximal neck length [15].

We performed EVAR in this complex case despite failed attempt at an outside hospital. Short-term outcome was satisfactory without endoleaks or stent-graft migration. Occlusion of the internal iliac artery (IIA) during AAA repair has been associated with buttock claudication, impotence, colon ischemia and pelvic necrosis [16]. But with regard to aneurysmal dilation and severe calcification of bilateral iliac arteries in this case, bilateral IIAs occlusion was considered in order to reduce the potential risk of endoleaks and future rupture. Pavlidis et al. reported that buttock claudication was a frequent complication after interventional occlusion of internal iliac artery, which often persisted during follow-up [17]. Alternatives such as iliac side-branched graft or sandwich technique maintained pelvic perfusion should be considered. However, these adjunctive procedures inevitably increase the risk of endoleaks and technical difficulties, especially under local anesthesia. Fortunately, we didn’t find significant complications in this case during follow-up, otherwise open repair maintained perfusion to IIA would be suggested.

As far as we know, no data exist on direct comparison of the performance of different stent-graft type in EVAR for AAA, and optimal selection of stent-graft type in hostile neck remains unclear [9]. A retrospective review of patients undergoing EVAR with unfavorable neck anatomy using the C3 Excluder repostitionable stent-graft demonstrated favorable short-term results and significantly reduced the need for proximal extension cuffs [18]. Bastos et al. also reported satisfactory early results using the Endurant stent-graft system in severe proximal neck angulation and no sealing length was lost in extremely angulated cases, confirming the device’s high conformability [19]. In addition, Perdikides et al. and Albertini et al. reported that endovascular repair using a flexible Aorfix stent-graft was feasible in patients with highly angulated necks and mid-term results were acceptable [12,20]. Considering first failure of EVAR with Endurant graft-stent from left access, we decided to use another flexible Excluder stent-graft from right access in this difficult case. Highly conformable devices (such as the Excluder) can adapt to the underlying anatomy, and reduce the displacement forces on the graft and avoid the gaps that originate type I endoleaks. Intraoperative angiography and 6 months follow-up CTA demonstrated a complete sealing of AAA without stent-graft endoleaks and migration, but life-long follow up is still needed.

Beside hostile proximal neck anatomy, challenging artery access conditions such as small-caliber vessels, iliac tortuosity, excessive calcification and occlusive diseases, represent the second most common excluding factor for EVAR [8]. Technical developments including downsizing lower profiles, improved flexibility of the stent grafts and wire techniques (stretch guidewire technique and buddy wire technique) expanded the application of EVAR. In this case, tortuous aorta and aortoiliac access made the insertion of the stent-graft delivery system very difficultly and the wire access to left iliac limb was unable to be obtained through the routine access from femoral artery. Therefore, we proposed a snare to create wire access from right radial artery to left femoral artery which ensured left iliac leg through tortuous anatomy, thus decreasing procedural time and radiation exposure. Of course, several other maneuvers might be adopted in this particular situation. Buddy wire technique can straighten iliac artery and provide support to facilitate stent-graft deployment but should be cautiously used for severe calcified artery. Balloon-assisted technique can better anchor the wire while advancing the sheath through the tortuous path which eased guidewire engagement [21]. However, the balloon blocking the aorta in the technique brings greater circulation disturbance and higher risk of visceral ischemia.

## Conclusion

EVAR in AAA with severely angulated neck and tortuous access is technically challenging. Choosing flexible stent-graft system and various alternative techniques may make the difficult cases feasible, and achieve an encouraging early outcome. Further long-term randomized studies are needed to confirm the safety and durability of EVAR in patients with hostile anatomy.

### Consent

Written informed consent was obtain from the patient for publication of this case report and any accompanying images. A copy of writen consent is available for review by the editor of this journal.

## Abbreviations

EVAR: Endovascular aneurysm repair
AAA: Abdominal aortic aneurysm
CTA: Computed tomographic angiography
